# Involving Citizen-Patients in the Development of Telehealth Services: Qualitative Study of Experts’ and Citizen-Patients’ Perspectives

**DOI:** 10.2196/10665

**Published:** 2018-11-12

**Authors:** Hassane Alami, Marie-Pierre Gagnon, Jean-Paul Fortin

**Affiliations:** 1 Institute of Health and Social Services in Primary Care Research Center on Healthcare and Services in Primary Care Laval University Quebec, QC Canada; 2 Research Center of Quebec City University Hospital Center Hôpital St-François d'Assise Quebec, QC Canada; 3 Faculty of Nursing Science Laval University Quebec, QC Canada; 4 Department of Social and Preventive Medicine Faculty of Medicine Laval University Quebec, QC Canada

**Keywords:** patient participation, public participation, health services, telehealth, health technology assessment, decision making

## Abstract

**Background:**

Decisions regarding telehealth services in Quebec (Canada) have been largely technocratic by nature for the last 15 years, and the involvement of citizen-patients in the development of telehealth services is virtually nonexistent. In view of the societal challenges that telehealth raises, citizen-patient involvement could ensure more balance between evidence from traditional research methodologies and technical experts and the needs and expectations of populations in decisions about telehealth services.

**Objective:**

This study aimed to explore the perception of various stakeholders (decision makers, telehealth program and policy managers, clinicians, researchers, evaluators, and citizen-patients) regarding the involvement of citizen-patients in the development of telehealth services in Quebec. In particular, we explored its potential advantages, added value, obstacles, and challenges it raises for decision making.

**Methods:**

We used a qualitative research approach based on semistructured individual interviews, with a total of 29 key actors. Respondents were identified by the contact network method. Interviews were recorded and transcribed verbatim. A pragmatic content thematic analysis was performed. To increase the capacity for interpretation and analysis, we were guided by the principle of data triangulation.

**Results:**

Citizen-patient involvement in decision making is perceived more as a theoretical idea than as a practical reality in health care organizations or in the health system. There is very little connection between citizen involvement structures or patient and user groups and telehealth leaders. For the respondents, citizen-patient involvement in telehealth could increase the accountability and transparency of decision making and make it more pragmatic within an innovation-driven health system. This involvement could also make citizen-patients ambassadors and promoters of telehealth and improve the quality and organization of health services while ensuring they are more socially relevant. Challenges and constraints that were reported include the ambiguity of the *citizen-patient*, who should be involved and how, *claimant* citizen-patient, the risk of professionalization of citizen-patient involvement, and the gap between decision time versus time to involve the citizen-patient.

**Conclusions:**

This study provides a basis for future research on the potential of involving citizen-patients in telehealth. There is a great need for research on the issue of citizen-patient involvement as an organizational innovation (in terms of decision-making model). Research on the organizational predisposition and preparation for such a change becomes central. More efforts to synthesize and translate knowledge on public participation in decision making in the health sector, particularly in the field of technology development, are needed.

## Introduction

### Background

Telehealth, defined as *health care and services, as well as social, preventive and curative services, delivered remotely by means of a telecommunication, including audiovisual exchanges for information, education and research, and treatment of clinical and administrative data purposes* [1], has become an inescapable part of health system reform strategies [2,3]. In recent years, many projects and programs have been initiated with the objective to develop new models of service delivery, capitalizing on the potential of telehealth to improve accessibility, quality, continuity, efficiency, and integration of health care and services, especially for populations with chronic diseases and those living in rural and remote areas [2,4-9].

To maintain its health system in a capacity to respond to population’s needs, while addressing the problems of shortage or unequal geographical distribution of health professionals, the province of Quebec (Canada) considers telehealth as a major lever [10,11]. However, telehealth raises several challenges, including e-literacy, confidentiality and privacy, patient’s data protection, and the medicalization of the living space, in addition to the symbolism that technology might endorse for some people or communities [12-15]. In the same vein, telehealth also raises issues related to digital democracy and the right of all citizens to equally benefit from advances made in digital health, which leads several authors to call for a better consideration of the perspectives of people and communities who are, or could be, affected by these issues [12,15-19]. To overcome such issues, at least in part, the idea of involving citizen-patients (the term refers to patients or their representatives, their family, as well as citizens, public, and communities who are actual or potential users of health services) in the decisions concerning the development of telehealth services has been proposed [19-23]. Moreover, this involvement should not only occur in the evaluation of telehealth as a *technical object* (eg, survey about technology acceptance and satisfaction), but in the prioritization, planning, and implementation of telehealth services.

### Public Participation in the Health Sector and in Telehealth

Public participation is attracting increased interest from health sector decision makers [24,25]. It has come, in a way, to compensate for the limits of the historically dominant technical knowledge from expert systems by giving more voice to the various actors from different knowledge sources [25]. This context is accompanied by a movement of institutional relocation of collective action for more consideration of different perspectives and opinions, which could be described as *lay*.

Public participation in decision making is also a way of narrowing the gap between evidence from traditional research methodologies and the expectations, real needs, and subjectivities of populations [26,27]. Thus, public participation could help to make informed decisions and reach a consensus (or at least a compromise), which would increase the legitimacy and scope of the policies and programs implemented [28].

In the field of eHealth, some studies report experiences of citizen-patients’ involvement. In the United Kingdom, citizen juries contributed to explore the barriers and facilitators to the implementation of eHealth services [22]. This study showed that citizens expressed a desire to be included in the development of eHealth and that their suggestions were taken into account by decision makers. Moreover, in the United Kingdom, volunteer delegates were recruited to form a panel of citizens to discuss the issue of integrating eHealth into health care services [19]. This study showed that citizens have a good knowledge of issues related to the use of new technologies and thought that their involvement in the development of eHealth programs would be very illuminating. Another study in Denmark explored the potential to involve the public in telehealth implementation [29]. The authors conclude that the success of telehealth depends strongly on the inclusion of the public in the process of planning and development of services. In Australia, members of a community have been involved in the development of a telehealth planning framework based on needs assessment. According to the authors, if telehealth is not progressing enough and is struggling to integrate the routine of providing health care and services, it is notably because it does not sufficiently consider the needs, priorities, and expectations of the communities [23]. In fact, this perspective would provide an opportunity to reduce the tension between *universal and unbiased* assessment of the instrumental value of a technology and the values, judgments, and perceived needs of end users [30,31].

The relevance and necessity of considering the citizen-patient perspective in telehealth development have led us to question its feasibility in the context of Quebec. In this province, there is a will to involve citizens and patients in decisions that could affect their health, at least in the political discourse. In 2014, a report called *Clinical telehealth in Quebec: an ethical perspective* was produced to inform and sensitize decision makers, researchers, and the public on some ethical issues related to telehealth utilization [32]. This report emphasizes that telehealth should focus primarily on relevance and demand, not on the offer and technology development. Traditionally, decisions regarding telehealth in Quebec have been largely technocratic by nature, and the involvement of citizen-patients in the development of telehealth services is virtually nonexistent, except in some research projects. Therefore, many questions remain regarding the meaning, feasibility, and implementation of the citizen-patients’ perspective in the development of telehealth services.

### Objective of the Study

This study aimed to explore the perspectives of various stakeholders (decision makers, telehealth program and policy managers, clinicians, researchers, evaluators, and citizen-patients) regarding citizen-patient involvement in the development of telehealth services in Quebec.

Our primary interest was to understand the meaning of citizen-patient involvement in telehealth decision making in terms of potential advantages, added value, obstacles, and challenges it raises for decision making. In this study, we used the term *involvement* generically without focusing on any particular form. This choice allowed us to explore the notion of involvement in general and what it meant to the stakeholders.

## Methods

### Data Collection

We used a qualitative research approach based on semistructured individual interviews with stakeholders involved in, or affected by, decisions related to the development of telehealth services in Quebec. The interview guide covered dimensions related to the perception, added value, relevance, as well as the challenges of citizen-patient involvement in telehealth (Textbox 1). Most questions were same for all participants, but some specific questions were asked according to the status of the respondent. HA did all the interviews (face-to-face or by phone) in French and in a place that respected participants’ privacy. The interviews lasted between 45 and 120 min and were audio-recorded. None of the participants refused to be recorded. Participants received no financial compensation.

Potential respondents were identified by the contact network method [33]. For decision makers and managers, we contacted people through the network of our team that is active in the evaluation of telehealth programs and projects in Quebec. We contacted citizen-patients who had collaborated on some research projects in the past [33,34]. Internet searches were also conducted to identify other potential participants (*experts* and citizen-patients), particularly via government, organizational, corporatist, or associative documents related to telehealth. This choice was justified by the need to have data and information from various sources to cover the perspectives of different stakeholders. In addition, during the interviews, some participants also referred us to other people.

### Data Analysis

We performed a pragmatic content thematic analysis of the interview data [35-37]. Thematic analysis consists of identifying, classifying, and combining data to distinguish themes and to relate or integrate them with others [35-37]. The pragmatic dimension refers to the interpretative and emerging aspect of the data. Indeed, during the data analysis process, we used the comments of co-researchers or project-related people that could complement the analyses.

The interview transcripts were first read by HA who developed a preliminary coding tree. A research assistant independently coded 3 out of the 29 transcripts to propose, suggest, and add themes and delete or merge others if needed. This coding tree was then validated with the other researchers (MPG and JPF) to reach a consensus. To increase the capacity for interpretation and analysis, we were guided by the principle of data triangulation [38]. This was done at 2 levels: (1) methodological triangulation through the use of multiple data collection techniques (eg, semidirected interviews; informal discussions with researchers, policy makers, telehealth leaders, researchers, evaluators, and citizen-patients at conferences and symposia; or other events that occurred during the study) and (2) triangulation of data sources consisting of the search for information from various stakeholders [39,40]. The use of multiple techniques and data sources is recognized as being able to increase the credibility of the results [39,40].

We obtained ethical approval (number “2015-2016-18 MP”) from the ethical committee of the Research Center on Healthcare and Services in Primary Care of Laval University (Quebec, Canada).

**Interview questions (translated from French).**

*Experts* (eg, decision makers and managers, experts in telehealth and [or] in patient and public participation)

1. Could you give me your definition of telehealth?
2. Could you give me a portrait of telehealth in Quebec today?
3. According to you, how is telehealth developing in Quebec?
4. According to you, what are the reasons why existing telehealth projects are struggling to move beyond the phase of pilot project?
5. According to you, whose needs telehealth services are addressing? (Those expressed by professionals, organizations, or citizen-patients?)
6. According to you, what are the reasons why some citizen-patients express reluctance to be supported by telehealth?
7. Could you tell me about the way in which decisions are taken for the development of telehealth in Quebec (organizations, ministry)?
8. Did citizen-patients already express reluctance or comments about telehealth? If so, what suggestions or proposals did you make?
9. To your knowledge, are there already experiences of involvement of citizen-patients initiated by your organization on other topics (other than telehealth)?
10. How do you perceive the possibility to involve citizen-patients as leverage to better development of telehealth services?
11. How citizen-patients could contribute to the decision-making process to develop telehealth services?
12. How their proposals could be incorporated into the decision-making process?
13. What type of involvement would be more adapted to enable the development of services that are more focused on the needs and expectations of the population?
14. What kind of citizen-patient involvement would be more useful, depending on the level of involvement and its focus (strategic, operational, and clinical)?
15. According to you, how could this involvement be organized (should it be done within the existing decision-making structures, or should another one be created in parallel), why?
16. According to you, what are the advantages, benefits, constraints, and obstacles to citizen-patient involvement perceived by the decision-making authorities?

Citizen-patients

1. Could you give me your definition of telehealth?
2. According to you, how is telehealth developing in Quebec?
3. According to you, what are the reasons why existing telehealth projects are struggling to move beyond the phase of pilot project?
4. According to you, what are the reasons why some citizen-patients express reluctance to be supported by telehealth?
5. According to you, whose needs telehealth services are addressing? (Those expressed by professionals, organizations, or citizen-patients?)
6. Did you (or other citizen-patients) already express comments (enthusiasm or reluctance) about telehealth? If so, what suggestions or proposals did you make?
7. Were you already involved in the development of a telehealth services (or technological in general), or are you aware of citizen-patient engagement experiences in the development of technology projects (including telehealth)?
8. How do you perceive the possibility of involving citizen-patients as leverage for better telehealth development?
9. How citizen-patients could contribute in the decision-making process to develop telehealth services?
10. How could their proposals be integrated into the decision making?
11. What type of involvement would be more adapted to enable the development of services that are more focused on the needs and expectations of the population?
12. What kind of citizen-patient involvement would be more useful, depending on the level of involvement and its focus (strategic, operational, and clinical)?
13. According to you, how could this involvement be organized (should it be done within the existing decision-making structures, or should another one be created in parallel), why?
14. According to you, what are the advantages, benefits, constraints, and obstacles to citizen-patient involvement perceived by the decision-making authorities?

## Results

### Profile of Participants

A list of approximately 64 potential respondents was identified. In total, we were able to interview 29 people (Table 1).

The results are structured according to the following themes: (1) telehealth as technocratic or expert, (2) relevance and potential contributions of citizen-patient involvement in telehealth, and (3) challenges and constraints to involvement.

The codes used at the end of the quotes refer to the categories of respondents presented in Table 1. All interview quotes were translated from French to English.

### Telehealth as a Technocratic or Expert Object

All stakeholders recognize that telehealth decision making is dominated by a top-down and technocratic perspective. Here, we can distinguish 2 levels: (1) the central level, emanating from the Ministry of Health or funding agencies that have a major role in the prioritization and choice of telehealth services and programs to implement and prioritize the use of telehealth in some specialties, levels, or locations rather than others, and (2) the local level, consisting of projects that are more often initiated by clinical, managerial, and technological champions in collaboration with researchers, usually funded through some research budgets, and offer telehealth services, often on an experimental basis and responding to the needs identified by those champions. At this level, there are some attempts to involve patients, mainly to evaluate the usability (eg, ergonomics) of the technological device downstream of its design and implementation, but less about how the service is delivered or organized (eg, relevance of the service):

Are people happy with technology?...Because people can love technology but not like how it is organized.

M

Here, respondents recognize that there is very little connection between citizen involvement structures or patient and user groups and telehealth leaders. Thus, the involvement of citizen-patients in the development of services remains very anecdotal or nonexistent.

### Relevance and Potential Contributions of Citizen-Patient Involvement in Telehealth

#### Accountability and Transparency of Decision Making

The fact that decisions on public policy choices, including those regarding health services, need to be discussed, affordable, and understandable to the populations concerned has been reported regularly. Respondents believe that health care computerization policies involve issues that are important enough for individuals and communities to express themselves and be associated. As telehealth involves significant financial issues in terms of investments and expenditures for the health system, it is even more relevant to have a citizen-patient perspective that helps ensure accountability of decisions on such investments:

People will not all look for the Cadillac...There is also a question of simplicity and use. I think to put citizens, users around a table...It creates a minimum of obligation being to some extent transparent...than if it is only companies and healthcare providers who are together to choose the technology.

E

Citizen-patients could also contribute to help to implement relevant services and to remedy the current situation where technologies are developing without a real overview and sometimes exponential costs:

When I look at the innovations in which we will invest a lot: both public funds and private funds for 7 years, 10 years of development, and then at the end of the race, have a technology that sometimes, doesn’t always meet the needs, or doesn’t meet the most pressing needs, and I think that perhaps if, early in the design of innovation, we had better examined both the needs of clinicians and populations? I think we would have avoided...useless expenses and useless turns.

E

#### Pragmatic Decision Making and an Innovation-Driven Health System

Citizen-patient involvement was also seen as a means to influence and accelerate change and adoption of telehealth and integrating it into the health system. Here, reference is made to the repeated failures of telehealth and computerization projects in Quebec. According to some respondents, telehealth in Quebec today is associated with “it doesn’t work.” They estimate that leadership could come from the population, especially those living in rural and remote areas or living with chronic diseases. They can put pressure on organizations and decision makers and advocate telehealth as the center of priorities and strategic directions.

Tensions and conflicts between professional orders, unions, organizations, and the ministry regarding reserved acts, insurance, reimbursement, and remuneration issues accompany the use of telehealth. These challenges significantly contribute to the difficulties experienced by telehealth programs in Quebec today. This situation often leaves the right of access to services for the entire population as a *secondary objective*. Involving citizen-patients could help refocusing the debate on improving access, continuity, and quality of services for the population:

Well, but if the pressure comes from the population, in an environment where we say: “if we had such types of services in a region where there is a lot of diabetes, if we could treat like that, but we cannot because the union doesn’t want, you know”...or the worker or manager says: “I cannot. My union doesn’t want to,” you know...Oh well listen, me, what I think, sincerely...as long as the patient will not stand up and say: “I am tired. I’m not waiting anymore. There are technological systems that make me no longer have to wait or travel”...There is no counterweight. There, I think we touch the system the most...We touch the crux of the problem.

C

**Table 1:** Summary of interview participant’s characteristics (n=29).

Type of participants (category of respondents)	Participants, n	Gender, n	
		Female	Male
Decision makers (D)	4	2	2
Managers, technicians, and clinicians-managers (M)	11	9	2
Citizen-patient representatives (C)	7	2	5
Experts in telehealth and/or in patient and public participation, ie, evaluation + research (E)	7	3	4
Total	29	16	13

Respondents recognized that the involvement of citizen-patients would not only increase the awareness of decision makers and health professionals of the potential of technology but also shed light on its importance in people’s life. In addition, it would make decision making more pragmatic and rooted in the real needs and expectations and highlight the level of *acceptable risk* for individuals and communities. On this point, respondents believe that confidentiality and privacy requirements are rigidly addressed by the responsible authorities, which slows down the use of telehealth:

We, health system experts, have shown that we were unable to do it, and you know that it has been demonstrated, I think. And then, we even mentioned confidentiality reasons very, very often: “it is not safe; it’s not confidential...” It’s like if we didn’t include in the discussion those who are the main concerned by technology; that is to say, the citizen-patients themselves, because there is, in all this adventure, a risk that is never zero, but which was acceptable considering the benefits of technology. And it seems to me that the acceptable risk arbitration can only be made by citizen-patients and not by the health system actors. It’s a big mistake not to have associated them from the beginning so that these elements of acceptable risk can be addressed and discussed and decided by citizen forums...How far am I willing to take a risk that occasionally there is information that can circulate compared to the benefits it gives? Only the citizen or the patient can conclude on this acceptable risk.

D

For example, current safety standards and regulations still greatly limit the use of Wi-Fi networks in health care organizations or prohibit that clinicians communicate with their patients via common chat technologies. Some respondents acknowledge that despite all these restrictions, there are clinicians using these “unsafe” technologies to communicate and monitor their patients while knowing that it is legally prohibited because they estimate that the benefits are greater than the risks for the patient. In such cases, citizen-patients should be given the opportunity to estimate the *risk-benefit* and decide whether or not they want to use these technologies to communicate with their providers because ultimately, the information and data belong to them:

Me, if we had a FaceTime service, because I like FaceTime. I like to see who I am talking to (...) It would be nice if it was more with a visual contact for me and for many people (...) It improves the exchange, the exchange...I think, for a person who gets older, see the person you talk to [physician, nurse], if she has a smile, it’s like an encouragement...It’s reassuring, it’s encouraging, and there are many people who live alone as they get older and have not prepared for their old age. You know, it’s getting ready, loneliness.

C

This argument was also supported by the fact that people have to travel great distances, sometimes several hundred kilometers for a simple routine consultation that can last 10 min:

So, we had clinicians and also clients who wanted to use Skype...There were obstacles. For the clinician, it was just more convenient to communicate like this with the client at home, but because we were not in the standards of confidentiality, Skype was banned from the clinical services, but clients, they agreed to use it...they are agreeing and consenting. They want!

M

It was also reported that the rigidity of the health system and its difficulty in adapting to the trend of increasing use of digital technologies in people’s life, in addition to its inability to capitalize on the potential of these technologies in the production and the provision health care and services, could lead people to search for health services through unconventional means and channels, including digital platforms that offer Web-based health services, with all the risks that this might present for them. Respondents recognized that the possibility to have access to services quickly and cheaply could be attractive to some people. However, in cases where people are victims of medical errors or receive harmful prescriptions via these platforms, the health system will have to assume their care, sometimes with serious complications that would result in significant costs to the public. In addition, it was also admitted that the ability of some people to have rapid access to health services goes against the idea of an equitable and universal health system:

As long as there was nothing else than that, it was fine, but someone comes to offer something else, you know. And that was the Internet and the optics companies in the USA that sold...It’s the same as the taxi: “It doesn’t make sense; it’s illegal.” Well that’s what they say. Opticians still say it. OK? Well, people buy the glasses...Me, it’s striking what happened with taxis. Everyone thought taxis were fine. Overnight, someone who took “Uber,” he opens the door, the car is clean. Hey, that could be the taxi!

C

In this vein, respondents believe that the citizen-patient perspective could help managers and decision makers to be more innovative. This will make them more aware of the new uses of digital technologies and see how they could capitalize on it to improve services. On this point, participants acknowledge that there is a significant gap between what health organizations and health system are able to offer in terms of technology-based services and how people use technology today. It is feared that such a gap will continue to widen, particularly in view of the bureaucratic heaviness of the system:

In general, patients are very, very open and even wish to use ICTs [information and communication technologies], and it’s rather the health system that has reservations. When I look at how we can currently communicate with the health system, patients want to use e-mail, for example. While the system is very, very refractory; doctors are refractory; the Canadian Medical Protective Association warns doctors about this use. So, the obstacles are, in my opinion, much more at the level of the institution than at the level of the patients. It's very rare, patients...we see it with the Quebec Health Record...patients who have withdrawn their consent are extremely rare.

D

#### The Citizen-Patient as an Ambassador and Promoter of Telehealth

Many citizen-patients are more and more informed about health and technology. They are in the capacity to propose alternatives or service improvements. Some of them even do information monitoring on the latest technologies for a given service. They can advocate for technology with organizations and decision makers, as well as the community:

Me, I have a Facebook that is read a lot, and from time to time, I post. Here’s an application. People thank me “ah thank you, I’ll try it.”

C

Respondents considered that the citizen-patient can become an ambassador and promoter of telehealth services to the population. On this last point, there is a great ignorance of telehealth and its potential within the population:

Well, the word itself, I never heard that word. Heh no, me “telehealth,” I would have thought that it’s medicine classes that are given at the university. Honestly, I have never heard. Yet, I read the press and I think I am a pretty informed woman, and still the two committees where I am, I have never heard...Are there many people who use that?...First, we should talk about it...It would be wonderful.

C

In addition, another part of the population is still reluctant to use telehealth. There is also the idea that telehealth is associated with lower quality services or *poor medicine*, which pushes people to seek services in large urban centers. Respondents recognize that communication and pedagogy are necessary to explain and convince. They suggest that this could be done by people who had a positive experience with telehealth, sometimes better than professionals or experts. Thus, integrating citizen-patients in telehealth project teams would make them ambassadors to their families and communities. Respondents acknowledge that the voice of users is more credible and listened to by others, with more weight than that of professionals and decision makers in some cases. Their opinion can thus influence other users, positively or negatively, because they speak the same language and share certain experiences:

There is nothing like a doctor to talk to another doctor, well, there is nothing like a patient to talk with another patient.

M

On another level, some respondents reported that the citizen-patient could also be an ambassador of technology to health professionals, including doctors (advocacy). Examples have been reported of patients in rural areas asking their doctor to be consulted via telehealth while the latter was not using it:

This is an element that is very important and we, we live it and we have lived in some of our regions where the patient or the professionals tell the visiting doctor from the south: “can we do it by telehealth?” So, yes, there’s a huge lack of information. The population must be more and more aware to ask the doctor: “Can I do it by telehealth?” There are cases where we cannot and cases where yes, we can and we avoid moving the patient.

M

#### Relevant and Better Organized Services

Opinion, comments, and suggestions of citizen-patients have a significant weight with health organizations, clinical teams, and decision makers. Their feedback is in a way the *mirror* that reflects the relevance of the services offered to the population. For instance, in a telehomecare project, some patients have pointed out that they did not want to be “plugged in” the technology all day or on weekends; others asked that the service should be provided to them at particular times during the day, when the health professional could contact or consult them. These considerations lead to review and reshape the organizational model and adapt the service in the light of the reality of the patient.

#### Perceived Clinical Quality Versus Lived Quality

From a utilitarian perspective, some respondents also recognized that citizen-patient involvement in telehealth would reduce complaints received by professionals or organizations. This is a way to reduce the gap between the *perceived quality* by the health professional and the *lived quality* by the patient. Citizen-patients often lack the opportunity to express themselves. Involving them could be a means to gauge their satisfaction or dissatisfaction with services but also to make sure that technology does not create unrealistic expectations:

Because it’s me who handles the complaints in the organization and sometimes, we don’t understand between what the client wants and what we want for him. Sometimes, we want more for him or we want it differently. I think that if we had more client partners, we might better understand what they want and better adapt our services from the perspective of customer.

M

#### Social Relevance of Technology

Respondents underscored the importance of considering the cultural particularities, subjectivities, and social contexts of individuals and communities:

Especially in an Indigenous communities where the mentality is not the same. You know, you have to talk about culture. Culture is not the same. So, if you want your project to work, you are better off to join the community with you, because you may be rejected.

C

Thus, citizen-patients can challenge the ethical and societal aspect of telehealth and raise awareness about the subjective and lived experience of people. So, it is central to develop services centered on individuals and communities and better take into account the diversity of backgrounds, paths of life, family, social, and cultural contexts. This would limit potential derivatives of the technology (*technological solutionism*), with a tendency to the standardization of services. Technology may not be for everyone (“you talk to a TV...it’s scary for some. [E]”), recognizing the need for a better understanding of the conditions in which telehealth is useful or not, and for what type of population:

In palliative care...the nature of the needs is different...the nature of the care and how to provide it also. Just to care for people who suffer from dementia, when we question the family caregivers...to ask them what is the thing that would make a difference in your life? Do you know what they tell us? Is it baths? Is it respite?...Our main need is that you recognize us as a human person. How are you going to solve that with technology? Once again, it is the capacity to recognize the caregiver not as an instrument at the service of the demented person, but a human being who has needs...The rigidity of our programs makes that we can’t meet the real needs. How, through technology, can we make this happen? It’s a challenge.

D

Some respondents considered involving citizen-patients as necessary as it is urgent to think of telehealth as a philosophy (*societal question*) that challenges the ways of producing and providing services for the population. Such a change requires clear and transparent communication with those concerned so that telehealth can allow providing services differently but not with less quality:

Telehealth should bring a new philosophy of care; not a logic of support at any cost. It must also be logical that telehealth is there to make sure that people are more autonomous at home. Unfortunately, it’s more complicated, because we’re, again, in this kind of obsession to offer the same types of services, regardless of the tool we use; the same levels (...) It’s not a question of offering less services; it is to offer the service differently with another way to do it....Telehealth is not just a tool...It grows a distant vision of the care; it grows a delegated vision of care...It affects the empowerment of people to take care of themselves.

C

Respondents also mentioned that telehealth should avoid increasing the digital divide (eg, literacy and e-literacy) at the population level and consider people and groups without sufficient education, knowledge, or means to use it. Thus, involving citizen-patients would make it possible to refocus the priorities, relevance, and needs in decisions surrounding the implementation of telehealth services, often reduced to questions of norms, standards, and administrative issues:

Yes, but here, telehealth, and if someone doesn’t have the Internet at home, what are you doing with that... The rest of us, we have everything at hand; we will not ask the question. We say yes, it will work.

M

### Challenges and Constraints to Involvement

Despite the added value and perceived usefulness as well as the opportunities inherent to citizen-patient involvement in the development of telehealth services, the observation is that there is a lack of practical and concrete experience reported in health organizations or at the health system level.

#### Ambiguity of the Citizen-Patient

From the point of view of decision making, citizen-patient involvement is seen as the introduction of an element of uncertainty. Decision-making processes still remain structured and codified environments that share common referents, a common language, and converging visions. The addition of citizen-patients, who have their own values, language, and expectations as stakeholders in the decision making, makes it possible to question existing equilibriums that make decision makers and managers fear the loss of control over the decision.

Some respondents also raised the issue of decision-making accountability: Who is responsible for a decision made with the citizen-patient? What is the degree of responsibility of the latter? Thus, several questions emerge about the place of the citizen-patient in this new decision-making configuration. In this vein, respondents recognized that decisions in telehealth services are largely formulated at the higher level (eg, ministry or administrators), which leaves little room for maneuver to integrate this new actor.

In addition, the idea was raised that the citizen-patient can become an element of *triangulation* in delicate decision-making situations, where their role could be perverted to put pressure on decision makers, managers or on clinical teams, especially when there is a divergence in visions. Another issue that was raised is that citizen-patients could become spokespersons of the industry or consumer advocacy associations, in other words, *lobbyists*:

Patients who have dissatisfaction, who have something to say, do they deal with him? Is it likely to bring us to triangulation rather than people talking directly to managers?...How does it fit? How are you going to live? What are the case trajectories and in which cases will they deal with situations? In connection with the complaints commissioner too. That’s what you know, you have the users’ committee, you have the complaints and quality commissioner, and you have the patient...the person...I do not know how they can be called.

M

In the same vein, there is a risk that citizen-patient involvement is *symbolic* or even perverted to legitimize certain decisions without the people having really contributed, but whose presence could be used as validating such a choice.

Some respondents were reluctant about the idea of citizen-patient involvement. For them, the *fashion* of citizen-patient involvement meant that, for some decision makers and professionals, the discourse has taken over the development of instruments to do so. It is recognized as a major and rapid change that destabilizes all levels of governance. There is a scope to learn because of the significant change it brings in the work of decision makers and managers. Here, some respondents referred to incidents where information leaked during sensitive decision-making processes (eg, closing a rural emergency service) and where it took a lot of energy and time to calm down media, reassure municipalities, and communities. Such experiences made some decision makers very dubious:

How can we explain to a rural community that the retiring physician will not be replaced, and that the service will now be provided via telehealth from experts based in Quebec City or in Montreal?

D

#### A Complainant or Claimant Citizen-Patient

For some respondents, the involvement of citizen-patients in decisions was seen as confrontation. They considered them as mere claimants or complainants and not true partners or collaborators. Citizen-patient involvement is also perceived as slowing down the decision-making process:

To open the discussions to the citizens, to the patients? It’s not natural. Managers and decision-makers must be convinced that patients bring added value, and I’m not sure, at present, in Quebec in any case, that the majority of managers are convinced of this and, on the contrary, I think that they see the patient as a drag, an obstacle...in any case, something that slows down the process much more than a decision aid.

D

Some respondents also believed that the ambient discourse may fall into the caricature stating that citizen-patients are a virtuous source of good ideas at any times, hence the challenge of articulating the mechanisms to be able to produce the ideas, confront them, discuss, and question them publicly.

We must also be careful to not fall into excess and say that the citizens run the solution. That, I’m against...They can participate in the decision, but is not for them to make it.

C

#### Professionalization of Citizen-Patient Involvement

With the new role that the citizen-patient can, or will, have in decision making, the issue of the professionalization of citizen-patient involvement emerged, even pushing some people to question whether citizen-patients will hold a *professional title*, be overseen by union conventions, and compete for budget within an organization:

A patient representative spoke to someone at the Ministry and said, “The patient’s voice needs to be taken into account. We have to be involved in the decision” and the guy from the Ministry said “Are we going to put you in the Union?”...Me, I have already been told by a famous researcher that if we integrate patients into research projects, they will highjack research...The big question was what budget item are we going to put this in?

C

#### Decision Time Versus Time to Involve the Citizen-Patient

Decision makers work within a decision-making frame, often subjected to time and calendar constraints. Involving the citizen-patient would result in slow decision-making process, as it involves consultations and exchanges with an actor who does not necessarily have knowledge of the functioning of the health system. In fact, according to the respondents, from the moment the citizen-patient is involved, the process must be transparent and not only be stingy with information but also be concerned to transmit the right information in a suitable language, free of jargon and technical acronyms. In addition, the question of when and how would citizen-patient involvement be useful and necessary emerged:

I think it’s not a habit, first. Then, well, there may be an unwillingness to do so, because it makes the process heavier. Because we were looking for Mr. Everyone who may not understand the language, for whom we have to take time to explain. Maybe we have a vision of the result and we...to share with the client, it will be a too long process...Because it will delay time of implantation and things like that.

M

#### Which Citizen-Patient Should Be Involved?

The question of the *right* citizen-patient to involve was often mentioned and respondents pointed out the diversity of profiles, knowledge, opinions, and experiences of individuals and communities:

Then, you see, the citizen, in relation to technology, it takes citizens who are awfully informed to be able to understand. So if we think about citizen participation in developing, better documenting needs, acceptable levels of risk, it must be citizens who have been informed, to whom we are able to explain the issues and who are able to give us a point of view on it.

D

#### The Question of How

Many respondents rose the question on how to make the most benefits from citizen-patient involvement. Their main fear was that with increasing calls to involve them, it becomes more a tokenistic participation, so that decision makers and managers can say that they have associated the citizen-patient in their approach:

I tell you that with the patient partner, yes, it’s a beautiful concept, but how does it translate into real life, the recipe did not come with it....It’s fine in terms of diagnosis, but no one offered me instruments.

M

## Discussion

### Principal Findings

To the best of our knowledge, this study is the first to address the issue of citizen-patient involvement in decisions related to the development of telehealth services in the Quebec health system. It is also one of the few studies that explore this potential from stakeholders’ cross-perspective (decision makers, telehealth program and policy managers, clinicians, researchers, evaluators, and citizen-patients).

In this study, there is general agreement between the opinions expressed by *experts* and citizen-patients that telehealth decision making should further integrate the citizen-patient perspective. First, because such an approach, from a utilitarian and pragmatic perspective, would accelerate the adoption and diffusion of digital technologies in the provision of health care and services. In other words, involving citizen-patients would *push* organizations and the health system to be more innovative. This perspective, however, remains subject to criticism by the fact that citizen-patient involvement is reduced to a simple instrument that justifies the implementation of certain technologies that can have a harmful impact on the population (eg, with services of lesser quality for some individuals or communities). Second, citizen-patient involvement can constitute a major lever to build a health system able to offer services adapted to the needs, subjectivities, constraints, and real expectations of individuals and communities. This involvement could ensure that all citizens benefit from the potential of digital technologies in improving, maintaining, or restoring their health and well-being; limit the risk of possible drifts of technological progress; and force more accountability and transparency in decision making (eg, better organization of services, better quality of services, social relevance, and ethical issues).

However, some divergences were found in the discourse of *experts* and citizen-patients. Decision makers and managers have emphasized the operational aspect of this involvement. In this respect, they have raised several questions, among others: the profile of citizen-patients to be involved; the picture of a claimant, complainant, or lobbyist citizen-patient; decision time versus time to involve the citizen-patient; how and when to involve them in the decision making process; and their weight and responsibilities in the decision. Citizen-patients, for their part, have insisted more on the democratic aspect, which translates into the obligation for organizations and the health system to create a real space to better integrate their perspective into decision making, and that this involvement should go beyond the symbolic dimension. In addition, some citizen-patients, echoed by certain decision makers and managers, insisted that their involvement should not be intended to replace the work of decision makers and managers because the latter remain the ultimate people responsible for the decision.

Otherwise, our findings highlight the significant gap between the relevance and theoretical added value of citizen-patient involvement and decision making regarding the development of telehealth services in Quebec. Indeed, there is still an ambiguous perception and a certain caution toward the involvement of citizen-patients in decision making. As reported in this study, there are still many issues to be clarified, particularly regarding the taxonomy of involvement and the development and availability of concrete instruments and mechanisms to operationalize it. This observation leads us to consider that citizen-patient involvement in the development of telehealth services is still at the stage of *innovation* in decision making, both for conventional decision makers and for citizen-patients. Indeed, it is introduced into the actual decision model, mainly technocratic, where policy makers, managers, and (sometimes) clinicians are the only ones at the table. Thus, as an innovation, citizen-patient involvement should provide evidence of its relevance and added value for these actors.

Here, innovation means a set of new routines and working methods that aim to improve the results, efficiency, profitability, relevance, or experience of the actors [41]. It is also a set of practices, ideas, or objectives that are considered new by an individual, a group, or within an organization [42]. In telehealth, decision making takes place in a context of uncertainty, where several alternatives are possible, as solid evidence of efficiency, effectiveness, quality, security, and social relevance is still fragmented, incomplete, and sometimes contradictory or inconsistent [43,44]. In this context, taking into account the different available options and resources as well as the values, expectations, and needs of individuals, communities, and society as a whole, could lead to an *optimal* decision that is expected to increase the benefits while mitigating the risks to the population [45]. That being said, conflicts remain ubiquitous in any decision-making situation, especially when there is no single choice that is best for all stakeholders [46,47].

As an innovation, citizen-patient involvement in telehealth may be in competition with other existing practices, balances, dynamics, cultures (organizational and professional), and powers (or hierarchies), which could lead to a confrontation between different visions or conception of reality. Indeed, user acceptance also depends on their perception of how the innovation will affect them in their practice and the interactions that exist between the actors in the specific context [41,42,48]. Analyzing our findings through the lenses of the diffusion of innovations theory [41,42], we inferred that, first, different stakeholders were able to identify benefits and advantages of citizen-patient involvement in telehealth decisions (*relative advantage*). For them, this perspective could be relevant to the work to be done and improve relevance of decision (*tasks and activities*). Thus, several respondents who had experience with participation or had experiences as health service users saw the benefits. For others, the benefits are to be demonstrated, which is necessary to convince them (*observability*). Second, a major issue is the operationalization of citizen-patient involvement: how can it be adapted to find solutions that meet the needs and values of the actors involved, given the differences in current working methods and standards? (*compatibility*). In addition, a majority of respondents have never tested or experienced citizen-patient involvement before in decision making in their organizations (*trialability*). Third, citizen-patient involvement is still perceived by key stakeholders as complex to use and to implement (*complexity*). In fact, it is expected to have a high degree of uncertainty (*risks*), which would make it difficult to adopt and operationalize. In addition, for some respondents, there is a need of knowledge and instruments to properly involve the citizen-patient in decision making (*knowledge*).

On this last point, this study also showed that there is a problem of knowledge transfer and sharing of research results on citizen-patient involvement in the decision making. Some stakeholders still hold mixed or negative opinions about this involvement, such as power issues, management of complaints and claims, risks of blocking or complicating the decision-making processes (time and means required), and possibility of lobbying. However, the international literature reports a wide range of experiences and initiatives, involving both patients and the public, that can help inform decision making and make it more relevant: health care prioritization and health policy analysis [49,50], resource allocation and redistribution [51,52], services governance [53,54], and health technology assessment [55]. On this point, our findings support, to some extent, those of Chalmers et al that addressed the question of the actual use of research results in decision making [56]. In our situation, either this research is not really relevant to decision makers—which is not theoretically the case here—or there is a missing link between this and the decision makers concerned, which seems to be the case in our work. Therefore, it is important to focus on the issue of synthesizing, sharing, and transferring existing knowledge in terms of contribution and value added of citizen-patient involvement in decision making.

In addition, the issue of change management should be better addressed. Indeed, we noted that the resistance and reluctance of some decision makers and managers are more because of the ambiguity associated with citizen-patient involvement as well as the nature and importance of the changes, in particular of cultures and models, that it brings to decision-making processes. For example, respondents reported that their training does not cover this aspect and that they are not prepared for decision making with the public. Thus, the involvement of the citizen-patient as a new decision maker requires codifying and marking the process, better defining the concepts, and developing a clear taxonomy as well as ensuring the availability of necessary instruments (eg, implication strategy, training, toolboxes, and evaluation tools) to operationalize and integrate it into the decision-making routine. Future studies in Quebec, or in other similar jurisdictions, should establish a clear taxonomy of involvement to allow differentiation between the nature of the mandates, the levels (strategic, tactical, or operational), and the nature of involvement (eg, information and partnership) [57]. It is a fundamental step to better use the existing modalities of involvement and to adapt or develop others if necessary. Indeed, according to the literature, the relevance (even necessity), nature, level, and degree of participation depend on contexts, issues, projects, and interventions [31,58].

Moreover, there is also a need to clarify what voice to consider: citizen-patient or consumer [59]. Indeed, it is important to consider the emerging debate on the duality between *consumerist* (eg, consumer’s rights associations and consumer lobbying) and *democratic* discourses regarding the relationship of citizens and populations with public services, which has a strong impact on the nature of governance to be put in place [60].

Finally, in light of the challenges and questions raised by the omnipresence of digital technologies in the choices and priorities regarding the development and implementation of health services, the consideration of the citizen-patient perspective becomes unavoidable; this is regardless of how it takes shape. Indeed, digital health involves a number of societal choices and orientations that affect the values and the foundations of health systems: what role should digital technologies play in future directions? What are the inherent risks of using these technologies (equity, ethics, social relevance, and data governance)? Indeed, many challenges and questions related to the relation of individuals to technology are increasingly reported in the literature, such as quality of the services, clinical outcomes for patients, health and digital literacy, security and confidentiality issues, intrusion into the private life, medicalization of the living environment, depersonalization of the patient-clinician relationship, social and cultural relevance, and increase of inequalities on socioeconomic or geographic bases. In this respect, many questions are raised about the potential negative, intended or unintended, consequences of the use of information and communication technologies in health care (health-ICTs) on individuals and communities [12-15,32,61-66]. These questions can no longer be treated by experts within the health system in a way that is disconnected from the expectations and concerns of citizen-patients.

In this study, we have addressed the case of telehealth in the provision of care and services, but other issues that arise for countries, such as big data, social networks, robotics, artificial intelligence, nanotechnologies, personalized, and predictive medicine, would also require societal debates to find the best ways that these innovations can benefit the whole population, while keeping in mind issues of ethics, equity, and health democracy. Indeed, to improve the acceptability of the technology and its subsequent use, the expectations, concerns, and needs of the various stakeholders involved should be taken into account, making information available and transparent. On this point, it is recognized that one of the success factors of the implemented programs and policies would be a more active and explicit conception of expertise emanating from the experience of citizen-patients as well as their expectations [67]. However, it should be ensured that citizen-patient involvement is not only a simple pretext or medium that allows decision makers to justify their decision to implement technologies and services without any real consideration of the citizen-patients’ perspectives.

### Strengths and Limitations

This study explored stakeholders’ perceptions of citizen-patient involvement in the development of telehealth services in Quebec. Our findings highlight a number of points that could guide future works on the contribution of citizen-patients in the development of digital health for the production and provision of care and services in a manner that respects ethics, social relevance, equity, justice, and the protection of citizens.

The diversity of study participants allowed considering a wide range of opinions and perspectives about opportunities as well as challenges to be met before citizen-patients can be involved in telehealth decision-making process. In addition, the broad experience of the interviewees at all levels of decision making (policy, managerial, clinical, and technical) or as health system users increases the validity and reliability of our findings. Indeed, our sample made it possible to achieve saturation, diversification, redundancy, repetition, and stability of interpretations [37,68].

However, we recognize the limitations of the study. Given the exploratory nature of the study, we have selected *informed* individuals, including citizen-patients, who have a very good knowledge of the functioning of the health system and health-ICTs issues. This could have led to a convenience sample bias. Thus, our results do not necessarily represent the perspective of the whole population (eg, age, socioeconomic profile, and gender). In addition, the participation of only 7 citizen-patients, of whom only 2 were women, are also limitations of this study. Unfortunately, given the difficult context in which the recruitment took place, we recruited all citizen-patients who agreed to participate, regardless of their sociodemographic profile.

In addition, we also recognize that the timing of the study coincides with a major reform of the Quebec health system (the largest since 1971), which may have impacted the results. Indeed, many potential participants (managers and decision makers) could not be identified or joined because they changed positions or were unable to respond to our solicitation. Others had no visibility on the issue as they had just joined posts related to our research question. However, the particular context of the reform has been helpful in pointing out the gaps between the political discourse held in the reform, which calls for greater involvement of the public in decisions, and the reality of the actors, in the health organizations in particular, who are required to translate these directives on the ground. That said, our results could have been different in a nonreform context. Although our study was conducted in a single jurisdiction, the findings could possibly apply to other health care systems that are facing the same challenges regarding the need for more citizen-patient involvement in decisions and the blooming of digital health.

### Conclusions

In this study, we explored the perception of various stakeholders regarding the involvement of citizen-patients in the development of telehealth services in Quebec. Thus, the study provides a basis for future research on the potential of considering the citizen-patient perspective in planning and implementing telehealth services for a better alignment with the expectations, needs, subjectivities, and contexts of individuals and communities, while promoting a relevant and socially responsible integration of technological innovations into the health systems.

Our findings show that citizen-patient involvement in decision making is more perceived as a theoretical idea, carried as much by attractive idealistic and utilitarian discourses, than as a practical reality lived in organizations or in the health system. Here, there is a great need for research on the issue of citizen-patient involvement as an organizational and systemic innovation. The adoption of this new decision-making model with the citizen-patient would imply adaptations and adjustments by the various stakeholders concerned by telehealth, which is accompanied by changes and transformations in practices and cultures in organizations. Moreover, efforts to synthesize and translate knowledge on citizen-patient involvement in decision making in the health sector, particularly in the field of technology development, are needed.

## Abbreviations

ICTs: information and communication technologies
